# Computational estimation of calcium fluxes in isolated magnocellular neurons

**DOI:** 10.1186/1471-2202-16-S1-P299

**Published:** 2015-12-18

**Authors:** S Kortus, G Dayanithi, M Zapotocky

**Affiliations:** 1 Institute of Physiology of the Czech Academy of Sciences, Prague, Czech Republic; 2 Institute of Biophysics and Informatics, First Faculty of Medicine, Charles University in Prague, Prague, Czech Republic; 3 Institute of Experimental Medicine, Czech Academy of Sciences, Prague, Czech Republic; 4 INSERM U710/EPHE, Université Montpellier 2, Montpellier, France

## 

Current optical methods based on fluorescent indicators permit to measure the intracellular calcium concentration with a high temporal resolution. To analyze the physiological mechanisms that underly the calcium dynamics, however, knowledge of the calcium fluxes into and out of the cell is needed. Here we present a method that permits to separately estimate the influx and clearance rates, based on the measurement of Ca^2+ ^concentration during a series of depolarization-evoked calcium transients. We apply this method to investigate calcium clearance mechanisms in isolated magnocellular neurons of the rat supraoptic nucleus.

In the simplest case, we assume that the cytoplasmic Ca^2+ ^concentration during the transient is governed by a time-dependent influx *J*_influx_(t) through voltage-gated Calcium channels and by an outflux *J*_clearance _that depends only on the instantaneous calcium concentration [Ca^2+^](t):

(1) $$d[{\text{Ca}}^{2+}]/dt=J_{\text{influx}}(\text{t})-J_{\text{clearance}}([{\text{Ca}}^{2+}].$$

To separate the two fluxes, we first estimate the clearance function *J*_clearance_([Ca^2+^]). Near the end of the transient, *J*_clearance _dominates over *J*_influx_, and *J*_clearance _may be obtained directly as the measured Ca^2+ ^decay rate [1]. In contrast, near the peak of a transient the two fluxes are comparable, and *J*_clearance _therefore significantly exceeds -d[Ca^2+^]/dt. However, in this case the clearance rate obtained from a higher transient can be used as a good estimate. If the assumption of Eq.1 is satisfied, the clearance function is obtained as the envelope of the recorded return curves in the d[Ca2+]/dt vs. [Ca2+] plot. The calcium influx rate *J*_influx_(t) during each transient is then estimated by substracting *J*_clearance_([Ca^2+^](t)) from the measured rate d[Ca^2+^]/dt. We tested the adequacy of this procedure using surrogate calcium dynamics data.

For cells in which the endoplasmic reticulum (ER) noticeably contributes to the calcium transient [2], the clearance function *J*_clearance _is not solely dependent on the cytoplasmic calcium concentration, as was assumed above. In this case, the method described above can still be applied to experiments performed in presence of Thapsigargin or cyclopiazonic acid (CPA), to avoid release or uptake of Ca^2+ ^by the ER. Comparison of the estimated fluxes from the experiments with and without Thapsigargin/CPA can be used to investigate the ER-dependent calcium fluxes. We apply this method to freshly isolated magnocellular neurons, in which we used Fura-2AM to measure the cytoplasmic [Ca^2+^] during depolarization-evoked Ca^2+ ^transients of various amplitudes and durations; depolarization was induced [3] by changing the external K^+ ^concentration.

Our method of estimating the Ca^2+ ^fluxes may be used also in other cell types to help characterize the contribution of individual mechanisms to calcium dynamics.
